# Research in cancer needs pivotal changes

**DOI:** 10.1016/j.lansea.2024.100421

**Published:** 2024-05-10

**Authors:** The Lancet Regional Health – Southeast Asia

The WHO South-East Asia region reported 2,369,106 new cancer cases and 1,527,959 deaths in 2022. Compared with estimates in 2020, which *The Lancet Regional Health — Southeast Asia* discussed in a previous Editorial, new cancer cases have decreased by 5.15%. However, deaths due to cancer have increased by 5.78%, which is alarming. Cancers of the breast (313,537 cases), cervix uteri (195,898), and lung (185,636) continue to be the most-reported cancers from the region. Lung cancer caused the greatest number of deaths (166,290) followed by cancers of the breast (142,986) and cervix uteri (118,975). We need to act now to prevent these premature deaths given the evidence on some of the measures that have reportedly been effective in several settings.

Regional collaboration is important for development of region-specific recommendations for cancer prevention. The Asian National Cancer Centers Alliance has provided some wide-ranging recommendations on actions for policy makers across Asia. The preliminary key recommendations included breastfeeding, cancer screening, vaccinations against hepatitis B virus and human papillomavirus (HPV), reducing pollution and workplace hazards, avoiding alcohol, and promoting a healthy lifestyle (diet, healthy body weight, physical activity, and no smoking). Most importantly, we need strong political will to promote HPV vaccination and reduce risk factors such as tobacco use, alcohol intake, and air pollution in southeast Asia.

Breast cancer is the most frequent cancer among women worldwide. High-income countries report more cases of breast cancer than low-income and middle-income countries (LMICs). But LMICs have high inequities in cancer treatment due to high costs and lack of technical expertise. For example, a single-centre prospective study at Tata Memorial Centre, Mumbai showed out-of-pocket expenditure for breast cancer patients to be very high. Research on better strategies for hypofractionation (giving larger doses of radiotherapy in fewer fractions) is promising as hypofractionation is well-known to be cost-effective. Early findings of a single-centre randomised phase-3 trial by Yadav and colleagues in Chandigarh, India showed that for breast cancer, a 2-week schedule of hypofractionated radiotherapy with a 2-dimensional technique is better than a 3-week schedule. The *Lancet* Breast Cancer Commission published last month highly recommended early screening of breast cancer, especially in LMICs. Bhutan's strong political will is laudable as it implemented a national cancer screening programme in 2020 for cervical, breast, and gastric cancers with the involvement of multiple stakeholders and was able to cover more than 90% of the eligible population.

WHO recommends screening and early diagnosis as key strategies to control the high mortality. Artificial intelligence (AI)-based approaches are becoming increasingly successful in diagnosis and the findings are encouraging. Batra and colleagues did an interesting study to assess whether an AI-based predictive system would be able to detect lung nodules in CT images and predict the probability of the patient having a mutation in the *EGFR* gene. Such studies involving development of non-invasive and cost-effective diagnosis would help oncologists in identifying high-risk patients in low-resource settings. In another study, Gupta and colleagues assessed whether a deep learning model was able to detect gallbladder cancer in ultrasound images. The diagnostic performance of the deep learning-based approach was comparable to that of experienced radiologists. However, AI-based approaches need further validation by multicentre studies.

Under-representation of certain populations in research translates into inadequate care. For example, northeastern states of India are, unfortunately, not well-represented in cancer studies, despite bearing a high burden of cancer. Gao and colleagues analysed the clinical trials registered during 2007–21 in the Clinical Trial Registry-India. The authors found under-representation of clinical trials from northeast India in addition to fewer trials related to haematological cancers (compared with solid tumours) and head and neck cancers. Sathishkumar and colleagues followed up cervical cancer cases registered in 11 population-based cancer registries in India (2012–15) and estimated the 5-year survival. The authors found heterogeneity in the 5-year survival across the registries, with higher survival in urban registries compared with northeastern and rural registries. Furthermore, we need more studies and representation from ethnic communities. Zami and colleagues addressed this issue by including patients from the Mizo population (an ethnic community from the northeastern state Mizoram) in their study. The authors showed that overall survival was better than progression-free survival in patients from the Mizo population with head and neck squamous cell carcinoma. The authors recommended multimodality approaches over single modality approaches, which would lead to better survival.

Research funding bodies must encourage multicentre studies, as the findings would be more representative of the entire population compared with single-centre studies. Departments of Health must also track approved cancer drugs and monitor their overall clinical efficacy. Most tertiary centres are in urban areas, and accessibility becomes a challenge for people living in rural areas (particularly areas with difficult terrain) and people of low socioeconomic status. Increased funding for research on AI in drug discovery and diagnostics is the need of the hour, as low-cost alternatives may be able to reduce some of the inequities. There is large body of research indicating that a few modifiable risk factors (unsafe sex, air pollution, alcohol use and smoking) could be targeted to prevent high morbidity and mortality; hence, these risk factors must be a priority for national programme roll-outs.

*The Lancet Regional Health—Southeast Asia* is committed to advocating equitable care and control of cancer in the southeast Asia region. High-quality research is needed to inform policy and practice. Therefore, the journal invites randomised controlled trials, observational studies (cohort, case–control, cross-sectional), proof-of-concept studies, cost-effectiveness analyses, and meta-analyses related to cancer. Cancer deaths are preventable, and there is a pressing need for development of novel treatments, improvement of health-care access, and implementation of effective cancer prevention strategies at national and regional levels.

